# Building Bridges, Paediatric Palliative Care in Belgium: A secondary data analysis of annual paediatric liaison team reports from 2010 to 2014

**DOI:** 10.1186/s12904-018-0324-2

**Published:** 2018-05-22

**Authors:** Marie Friedel, Bénédicte Brichard, Christine Fonteyne, Marleen Renard, Jean-Paul Misson, Els Vandecruys, Corinne Tonon, Françoise Verfaillie, Georgette Hendrijckx, Nathalie Andersson, Ilse Ruysseveldt, Katrien Moens, Jean-Marie Degryse, Isabelle Aujoulat

**Affiliations:** 1 Institute of Health and Society- Institut de recherche Santé et Société (IRSS), Université catholique de Louvain, 30, Clos Chapelle-aux-Champs, Boite 1.30.13, B-1200 Brussels, Belgium; 2 Haute Ecole Vinci, Institut Parnasse-ISEI, 41, Clos Chapelle-aux-Champs, 1200 Brussels, Belgium; 3 Cliniques universitaires St-Luc, 10, av Hippocrate, 1200 Brussels, Belgium; 4 Hôpital universitaire des enfants Reine Fabiola, Equipe de liaison pédiatrique, 15, av. JJ Crocq, 1020 Brussels, Belgium; 5 Universitair Ziekenhuis Leuven, Department of Paediatric Hemato-Oncology, Kites-Team (Kinderen In Thuis En Supportieve zorgteam), Herestraat, 49, 3000 Leuven, Belgium; 6 Centre hospitalier régional de la Citadelle, 1, Bd du 12ème de Ligne, 4000 Liège, Belgium; 7 Universitair Ziekenhuis Gent, KOESTER - liaisonequipe Kinderziekenhuis Prinses Elisabeth, C. Heymanslaan, 9000 Ghent, Belgium; 8 Cliniques universitaires st Luc, Interface pédiatrique, 10, av Hippocrate, 1200 Brussels, Belgium; 9 Scientific Institute of Public Health (ISP-WIV), 14, rue J. Wytsman, 1050 Brussels, Belgium; 10 Katholieke Universiteit Leuven, Oude Markt 13, 3000 Leuven, Belgium

## Abstract

**Background:**

Although continuity of care in paediatric palliative care (PPC) is considered to be an essential element of quality of care, it’s implementation is challenging. In Belgium, five paediatric liaison teams (PLTs) deliver palliative care. A Royal Decree issued in 2010 provides the legal framework that defines the PLTs’ missions, as ensuring continuity of curative and palliative care between the hospital and home for children diagnosed with life-limiting conditions. This national study describes how PLTs ensure continuity of care by describing their activities and the characteristics of the children they cared for from 2010 to 2014.

**Methods:**

Thematic analysis of open-ended questions was performed and descriptive statistics of aggregated data issued from annual reports, collected by the Belgian Ministry of Public Health through the Cancer Plan was used. A review panel of PLT members discussed the results and contributed to their interpretation.

**Results:**

Between 2010 and 2014, 3607 children and young adults (0–21 years) were cared for by the 5 Belgian PLTs (mean of 721/per year). Of these children, 50% were diagnosed with an oncological disease, 27% with a neurological or metabolic disease. Four hundred and twenty eight (428) children had died. For 51% of them, death took place at home. PLT activities include coordination; communication; curative and palliative care; education; research and fundraising. Different perceptions of what constitutes a palliative stage, heterogeneity in reporting diagnosis and the current lack of specific valid indicators to report PPC activities were found.

**Conclusion:**

PLTs are offering highly individualised, flexible and integrated care from diagnosis to bereavement in all care settings. Improvements in data registration and implementation of outcome measures are foreseen.

## Background

Children with life-limiting or life-threatening conditions require paediatric palliative care (PPC) to various degrees and intensity. The World Health Organization and the European Association of Palliative Care define activities of PPC as “the active total care of the child’s body, mind and spirit, (as well as) giving support to the family. It begins at diagnosis, and continues regardless of whether or not a child receives treatment directed at the disease. Healthcare providers must evaluate and alleviate a child’s physical, psychological and social distress. Effective palliative care requires a broad multidisciplinary approach that includes the family and makes use of available community resources” [1].

A recent cross-sectional analysis conducted in 23 countries revealed that 11 million children warrant a PPC approach, with 8 million of them needing access to specialised PPC services [2].

Various types of PPC services have developed over the past 20 years in Europe. These can be classified into three main categories: hospital-based; freestanding facilities or home-based services. To date, it is not known which one of these services best ensures continuity of care [3].

Continuity of care is a fundamental part of PPC because of the high number of health professionals the family is in contact with, the various care settings involved (in and out of hospital), and the significant distress experienced by the child and his/her family at different stages (illness, death, bereavement). From the perspectives of families, continuity of care is seen as a “seamless” organisation of care [4] and is a major element of quality palliative care. What remains unclear is how continuity is defined and what are the enablers and barriers to continuity of care in PPC.

Haggerty defines 3 types of continuity: informational continuity, management continuity and relational continuity [5]. Informational continuity involves the delivery of relevant information in a timely manner. Management continuity involves sharing consistent and adaptable goals across care teams and between patients and their carers. Relational continuity refers to a therapeutic relationship with one or more health professionals over time.

Further definitions of continuity of care can be found in the multicomponent model described by Freeman [6, 7] and reported as follows by Parker: “Continuity was defined as the experience of a co-ordinated and smooth progression of care from the service user’s perspective (experienced continuity), dependent on services that had: excellent information transfer (continuity of information), effective communication between professionals and services, and with patients (cross-boundary and team continuity), the ability to be flexible and adjust to the needs of the individual over time (flexible continuity), one or more named individual professionals with whom the patient could establish and maintain a therapeutic relationship (relational or personal continuity)” [8].

Saultz calls relational continuity the interpersonal continuity of care [9]. This is characterised by a trusted relationship between care providers and patients. Similarly the concept of continuing bonds is found to be critical in a PPC context, [10] where patients need to be cared for long periods and are in contact with many different health professionals in several care settings. Continuity of care can be achieved through nurse-led care coordination or a nurse case manager offering individualised, family-centred care [11, 12].

Inherent to these definitions and according to international recommendations is that PPC should start at the time of diagnosis of a life-limiting disease [1, 13]. More recently, the World Health Assembly called for governments to integrate PPC within a continuum of care promoting quality of life [14].

Early referral to PPC services is challenging [15]. Cultural barriers and organizational hurdles must be identified and overcome. Whilst there is a lack of descriptions of and comparisons of models in PPC, indicators of continuity of care have been established [11, 16–20].

These include the following characteristics: family-centred; accessible 24 h/day care; coordinated between in-and out-patient settings; delivered by an effectively communicating interdisciplinary team and including the possibility to access respite care and bereavement support.

### Context of Paediatric Palliative Care in Belgium

The first paediatric home care service was initiated in 1989 in Gent (Flanders) as a mobile team originating from the Oncology Department of a university hospital with the objective of providing curative care to children suffering from cancer. The aim was to reduce visits to the oncology day hospital. This team accepted the challenge to ensure continuity of care for children requiring palliative care who wished to return home.

The Belgian Act on Palliative Care (2002) protects the rights of all patients to receive comfort care in case of an incurable disease, [21] after disease-oriented therapies have failed.

In Belgium, PPC is offered through 5 specialised paediatric liaison teams (PLTs) affiliated to 5 university hospitals. Two are located in Flanders, 2 in Brussels (capital) and 1 in Wallonia. In 2010 a Royal Decree [22] stated that continuity of care should be available for in- and out-patients with serious illnesses (according to the 4 groups defined by the Royal College of Paediatrics and Child Health in 1997) [23] and categorised into 3 different groups of care: curative care, palliative care and terminal care at end-of-life stage.

Standards for accreditation requirements include the affiliation to a hospital which treats a minimum of 50 new patients < 16 years of age per year suffering from a haemato-oncological or haematological non-oncological illness which requires complex care, such as a stem cell transplantation. Furthermore, the Royal Decree foresees an interdisciplinary team with 4,0 Full-time equivalent (FTE) nurses, 0,5 FTE psycho-social care worker, 0,5 FTE physician and 0,5 FTE administrative assistant. Tandem work is required with the so-called first line, considered a primary health care team including a GP and home care nurses.

The liaison teams were first financed by “Kom op tegen Kanker” (a cancer charity) and by private funding. Since 2006, they have been predominantly funded by the Belgian Federal Government of Health with funds that were initiated by action point 23 of the Cancer Plan [24]. Since 2012 this action point is used to further develop and expand the function of a paediatric liaison team and its accompanied tasks. Financial funding is allocated according to the number of children cared for.

Based on the data collected through annual reports and addressed to the Belgian Federal Government of Health, this study aims to (i) describe the characteristics of children cared for by PLT, (ii) the different activities provided by PLT in order to document how continuity of care is ensured in Belgium. To the best of our knowledge, it is the first publication describing the characteristics of children cared for by the paediatric liaison care teams and the activities they deliver to families, offering important insights on the number and the characteristics of children accessing paediatric palliative care services in Belgium.

## Methods

### Study design

This study is a secondary analysis of the data provided by the 5 PLTs in the form of their annual reports and data collected by the federal government. Preliminary findings were presented to the PLTs and thereafter enriched by their nuanced interpretation of the results.

### Data collection

Aggregated quantitative data and narrative responses to open-ended questions issued from the annual reports 2010–2014 were transmitted to the first author. The data had been extracted from an existing database and is collected by the Cancer Plan then stored at the Belgian Federal Government of Public Health which holds the results of standardised annual PLT activity reports from 2010 to 2014. Annual reports have been mandatory since 2010, the year of the Royal Decree pertaining to the role of PLTs. The head nurse of each PLT completed the annual activity reports and sent them to the Cancer Plan.

The variables required for annual reports are presented in Table 1 and include the number of children cared for, their pathologies, the length of follow-up, as well as the number and places of deaths (hospital or home).

**Table 1:** Variables for aggregated quantitative data and open-ended questions included in the annual activity reports of the paediatric liaison teams

Age range classification	0–1 Y, > 1–10 Y, 11–20 Y, > 20 Y
Disease classification	Haematology/Oncology
	Neurology/Degenerative
	Genetic/Metabolic
	Cardiovascular
	Gastroenterology
	Nephrology
	Congenital abnormalities
	Immunological
	Respiratory
	Others (Neonatology, Transplantation)
Origin of referral Classification of patients Number of patients	Origin of referral for new patients (same hospital/other hospital) Curative/palliative/end-of-life patients Total patients followed-up per year
Death	Place of death (home, hospital, other) Total number of deaths Duration of follow-up Frequencies of admission in hospital Mean average duration of admission Number of contacts with families post-death
Objectives and needs	What objectives did you have? Which objectives did you achieve? Which objectives did you partially achieve? Why? What are your needs? Which objective(s) did you not achieve? Why? What are your objectives for next year? Did your team change during this year?
Strengths and weaknesses	What are the strengths of your team? What are the weaknesses of your team?
Needs assessment	What are the requests of the target group? What are the requests of the PLT? What are the requests of services/institutions/hospitals?

### Data analysis

Firstly, the characteristics of the children cared for by the five PLT over a five-year period, were summarised, using descriptive statistics.

Secondly, in order to identify and describe the various activities carried out by the PLT as part of their work, responses to the open-ended questions included in the annual reports were analysed by the first author inductively, ie. without any preconceived idea or hypothesis, to identify the themes that would best describe the range of activities undertaken by the PLTs [25].

Finally, the relevance and accuracy of the themes derived from the analysis performed by the first author were discussed with the representatives of the PLTs, as well as the Cancer Plan. The latter was consulted during a formal expert review panel meeting, held on 12th April 2016, which included 4 paediatric liaison nurses (CT,GH,FV,NA); 2 specialist physicians supervising the liaison teams (MR, CF); 1 social worker (IR) acting as a coordinator of a PLT and 2 researchers (IA, MF).

During this meeting, six themes were validated as describing the range of activities undertaken by the PLTs: coordination; communication; care; fundraising; training; quality improvement and research.

Moreover, several challenges encountered by PLT emerged in the annual reports and were discussed during the meeting. Those specific challenges will be presented in the results section.

The results presented hereafter, are therefore the expression of a collaborative approach of data analysis and interpretation, which involved the main stakeholders with the aim to collectively engage in a process of reflecting on past, present and future challenges to improve continuity of care in PPC.

## Results

### What are the characteristics of the children followed?

Based on the aggregated data from the annual reports for the period 2010–2014, a total of 3607 children in Belgium benefitted from a follow-up of one of the five PLT (average of 721 children per year). Among all the children >followed, the proportion of palliative patients was 25% (*n* = 910). The mean duration of follow-up was 226 days per child. Of the 3607 children, 47% were > 1 and < 10 of age, 25% were < 1 year, 25% were > 11 and < 20 years and 3% were > 20 years.

Fifty percent suffered from an oncological pathology, 27% from neurological, genetic abnormalities, metabolic or degenerative diseases and 23% consisted of others diseases (including cardiovascular, immunological, gastroenterological, respiratory, nephrological or perinatal diseases).

Four hundred and twenty eight children (12%) died during the study period. 51% percent of these children died at home, 47% died in hospital and 2% died in respite care services or institutions for disabled children. Each year, more than 40 home deaths were coordinated by PLTs across Belgium.

The characteristics of the children followed from 2010 to 2014 by the PLT are presented in Table 2.

**Table 2:** Characteristics of the children followed from 2010 to 2014 by the paediatric liaison teams (*n* = 3607)

Characteristics of children	Number of children (%)	Mean per year/per team (min/max)	Median per year/per team (P25/P75)
Age of children			
0–1 y	885 (25%)		
1–10 y	1689 (47%)		
11–20 y	879 (24%)		
> 20 y	116 (3%)	144 (43–240)	144 (103–196)
	Total of children: 3607 (100%)		
Pathologies			
Haematology/Oncology	1816 (50%)		
Neurology-/Degenerative/Metabolic	989 (27%)		
Other (Neonatology/Gastroenterology/Respiratory)	849 (23%)		
	Total: 3654 (100%)		
Curative/Palliative Patients			
Curative patients	2540 (70%)	102 (1–181)	105 (82–135)
Palliative patients	910 (25%)	36 (10–86)	23 (20–58)
Undefined	157 (5%)		
	Total: 3607 (100%)		
Referral of new patients			
From originating hospital	1848 (51%)		
External hospital	108 (3%)		
Total new patients	2056 (54%)		
	Total: 3607 (100%)		
Length follow-up (children who died)		226 days/child (1–4612 days)	32 days/child (21–201 days)
< 120 days	272 (64%)		
121–240 days	65 (15%)		
241–400 days	31 (7%)		
> 401 days	60 (14%)		
	Total: 428 (100%)		
Location of death			
Hospital	200 (47%)	8 (2–22)	6 (6–10)
Home	221 (51%)	9 (1–22)	9 (3–16)
Others (respite care home, others)	7 (2%)	18 (3–32)	19 (12–26)
	Total: 428 (100%)		

### What are the main activities of PLT?

The thematic analysis of the answers given by each PLT to the open questions in the annual activity reports, enriched through discussions with the review panel, resulted in the identification of 6 main types of activities, which are presented hereafter with quotes from the annual reports (AR) or from the review panel meeting (RP).

#### Coordination

Coordination between home care services, hospital services, schools and respite care services was reported as an important activity. Early referral to a PLT allows the team to build relationships with the families. The referring hospital-based-specialist physician continues to be responsible for the care of the patient until death. Interdisciplinary meetings within hospital units and out-patient services are initiated and coordinated by PLTs.

In order to enhance coordination reports of home-visits and interdisciplinary meetings are stored as electronic records. The phone calls made are recorded in “liaison notebooks” which are kept at the home. These notebooks can be used by the child, family members and caregivers to write down their observations.

Several difficulties in coordination were identified by PLT. These are linked to the absence of a social worker or a permanent coordinator, but also sometimes to the lack of a general practitioner identified by the family, which is a condition to the initiation of PPC home care.

PLTs express “that *the first line is established better in Flanders (northern Flemish speaking-part of Belgium) facilitating therefore the identification of a GP and home care nurses, which seems occasionally, to be a problem in Wallonia (south French-speaking part of Belgium).”* (RP).

One team has created the role of a “coordinator of information”, who is based permanently in the hospital to share the information among all health professionals, both for in-hospital and out-patients, and with families. “*This new function represents a valuable support for PLT.”* (RP).

Some PLT developed not only PPC home-based care, but also in-hospital PPC consultations, depending on institutional support and sometimes person-related factors.

*“It depends on certain individuals in the hospital whether or not your PLT is recognised. This has consequences for early referral. But I must say; now it’s better than before. The other in-hospital services know who we are and what we do.”* (RP)

#### Communication

Raising awareness among hospital teams, home care services and general society is achieved through brochures, conferences and fundraising, because PPC is still perceived as being limited to end-of-life care. The term “palliative” frightens the families and may represent an obstacle to accessing palliative care services.

*“Sometimes the families do not even know that we are the paediatric palliative care team. We are called liaison teams and introduced sometimes as a supportive team by the physicians.(…)The word ‘palliative’ frightens families.(…) Some families consider us as ‘the angel of death’, even though we know very well that palliative care is not restricted to end-of life care.(…) Right from the beginning, we should explain clearly, what the term palliative care means and repeat it as often as necessary!”(RP)*

Teams express the urgent need to represent and advocate for PPC through active membership at federal commissions and associations for palliative care.

*“I have been involved in the Palliative Care Federation of Flanders as an administrator since 2015 and am also the delegate in the Cell of Federal Assessment of Palliative Care (first meeting in 2015).” (AR)*

#### Care

PLTs provide complex individualised, patient-centred curative and palliative care at home or in hospital settings, when home based care teams are not available or not trained enough. Families can call and receive home visits at any time (available 7 days/24 h). PLTs have observed progressively more complex chronic conditions in children resulting in increasingly higher burdens for the family, who in turn require additional social assistance, financial resources, support for administrative procedures and in-home respite care services.

Coordination for complex procedures such as chemotherapy, blood transfusion, assisted ventilation, feeding through gastrostomy, tracheotomy care, patient controlled analgesia and intravenous sedation, requires constant professional training.

*« Liaison teams are small teams where patient care is the priority; the patient population is very diverse often with very complex issues. Delivering care takes time and is very intensive.» (AR)*

Care continues even after a child’s death. For each family, who wished it, bereavement care was offered via telephone, home visits or letters up to 1 year after the child’s death. In 2014, for example 672 contacts were established for 99 families who had lost their children (mean of 7 contacts/family).

#### Fundraising

Concerns regarding lack of resources were repeatedly expressed by the PLTs. Lack of trained PPC nurses in first-line home care services is also reported. One team states they do not even have an office. For other teams, a full complement of staff members to be able to ensure bereavement care, psychological support for siblings or a supervision of PLT is only possible thanks to private donations.

Monthly, mandatory supervision sessions are offered via an external psychologist. All team members can suggest some themes they want to discuss. Usually, debriefing is provided for challenging clinical situations. The main objectives of these sessions are to promote quality communication, to prevent compassion fatigue and assist team members to process vicarious trauma they may experience.

Lack of adequate governmental financing was repeatedly mentioned as obliging teams in time-consuming activities to find additional private funding to cover the total operating costs of the team. Including a social worker and a coordinator in the team was particularly emphasised.

*“Strengthening the team by the presence of a social worker (halftime) would free up time spent on many serious social tasks.” (AR)*

*“A special fundraising was undertaken for the provision of a vehicle.” (AR)*

*“We would like to offer regular bereavement care for siblings, but we lack funding. It’s a pity, because siblings are really overlooked.” (RP)*

*“We continue to seek additional help (when lack of personnel) through our own members’ networks.” (AR)*

#### Training

Continuous training for the team itself regarding pain control, paediatric palliative care, and complex care techniques such as intravenous chemotherapy or blood transfusion is carried out. When a new nurse enters the PLT, a long training period is offered for peer training; integration of care delivery; organisational elements of services; awareness and knowledge of key contacts. Thus acquiring competences in PPC needs time due to the complexity of the interventions provided.

Teams also ensure specific child and family counselling, the education of first line home care services who are not always specialised in paediatrics (for e.g. handling a gastrostomy, patient controlled analgesia pump, insertion of a nasogastric tube) as well as various professionals in hospital settings. Tailored support for schools around issues related to bereavement is also provided. Many PLT members are offering PPC education in nursing schools, faculties of medicine or hospitals.

*“We should implement or reinforce training in paediatric palliative care for physicians and nurses of the 1st line.” (AR)*

#### Quality improvement and research

Teams are aware of the importance of quality improvement, and are keen to establish workload indicators and patient-reported outcome measures (PROMS). Caring for patients limits the teams’ ability to engage in quality improvement and research activities. This was a source of frustration for health professionals.

*“These projects are carried out with a minimum of resources and staff and rely heavily on volunteers although our Government imposes these indicators and record keeping. The same sector attaches great importance to the quality indicators and recording because it improves the professionalisation of care and the development of care is factual.” (AR)*

Protocols on several themes are elaborated in different ways in each PLT. Some create flow-charts to determine feasibility of PPC home care, others create protocols on how to respond to a euthanasia request, should it occur, or how to implement Advanced Care Planning tools.

*“Palliative care, but especially early advanced care planning should be implemented in a better and more structurally sound way in paediatric services. Advanced care planning is very important in the care pathways for children with chronic conditions. This would fight therapeutic obstinacy; place care on a different and broader perspective, and allow parents / families to participate in the implementation of care goals. In paediatric palliative care, advanced care planning is an essential part of the care for children with chronic complex diseases. This still must be implemented.” (AR)*

### What are the specific challenges faced by PLTs?

In addition, during the review panel held in April 2016, the representatives of the 5 PLTs asserted that the workload of their teams was not truly reflected through the data reported in the annual reports. PLT’s highlighted 3 difficulties. These involved i) the way of reporting on cases, ii) the criteria to define when a palliative stage starts and iii) when to end a follow-up.

Firstly, with regards to reporting on cases, children followed by the PLTs present, over time, more complex chronic conditions, defined as medical conditions that can last at least 12 months and involve either several different organ systems or one organ system severely enough to require specialty paediatric care and probably a period of hospitalisation in a tertiary care center [26]. The PLTs expressed their difficulty to identify the principal disease of the child and in which category or categories of illness it should be reported.

Secondly, PLTS expressed difficulty in distinguishing patients requiring palliative care: “When curative treatment is no longer an option», «when death approaches”, “when the illness isn’t curable anymore and will lead to a premature death. But this does not mean that the patient doesn’t receive an illness-directed therapy. It has already happened that a patient labelled “palliative” shifted to “curative” because an experimental therapy succeeded. But for patients with neurometabolic illnesses, with a slow disease progression, the patient arrives at a palliative stage later on, when his quality of life deteriorates. It’s very different.”

This difficulty expressed by PLT to define when palliative care starts and actually stated in the annual reports, is significant and could be explained by the principle role played by PLT in being a seamless service starting at the time of diagnosis and offering a progressive step by step provision of PPC. Further, a distinction between patients receiving palliative versus curative care did not affect service provision. This discussion led to the reflection on whether a formal distinction between those two stages is required.

Finally the review panel expressed difficulty in determining the criteria by which a PLT should decide to end a follow-up. The duration of follow-up for patients can be difficult to determine, particularly when children become more stable and is variable depending on type and severity of disease, and family specific needs.

## Discussion

The results obtained in this national study showed that PLTs ensure continuity of care through a number of complex and complementary activities. More specifically, several results demonstrate how continuity of care is ensured, namely the long follow-up period (average of 7 months); starting at the time of diagnosis, often at a curative stage; the high number of home deaths; and the capacity to offer bereavement care to the family. Moreover, the difficulty for PLTs, to distinguish between curative and palliative stages, might indicate that those conceptual distinctions are not relevant to ensure continuity of care, whatever the child’s care pathway might be.

While other studies have reported a higher proportion of neurological diseases, [27–29] the large quantity of oncology cases in our study can be explained by several reasons. First of all, this is linked to the historical roots of the PLTs in Belgium that originated in oncology wards. Secondly the accreditation criteria defined by the Royal Decree, focusses on specialised oncology centres. Finally, the origin of funding for this programme, namely the Cancer Plan, likely influences the target population.

Regarding activities of the PLTs, our findings revealed a high number of home deaths, which requires an optimal degree of coordination among home care workers and hospital based professionals. It is commonly assumed that home would be the best place to care for a child at end-of-life, [30] but a study from Bluebond-Langner questions this assumption [31]. According to her, better outcome measures would be to assess the possibility for children and parents to have their preferences taken into account. This is the case for PLTs who offer families not only the possibility to express the location to have their child cared for at end-of-life (at home or at hospital), but also to rapidly adapt the place if preferences change over time.

Continuity of care such as provided by PLTs might be positively influenced through policies. In Belgium, considered among other European countries as offering optimal PPC provision [32], a bill voted in 2016 has enhanced the smooth integration of PPC within the national health system by enlarging the definition of palliative care beyond end-of-life care [21].

This is in line with the results of a systematic review conducted by Knapp et al. on PPC provision worldwide. Of the 43 European countries surveyed, 24 had some services in PPC. Belgium was one of only 5 countries providing the highest level of PPC services (level 4: measure of integration with mainstream services provided) [33].

Nevertheless, Noyes et al. demonstrate several potential barriers to accessing PPC services. Among these, cultural beliefs are highlighted as lowering the use of specialised PPC services [34].

Social acceptance of palliative care might influence optimal early access to specialised palliative care services and therefore continuity of care. One example is the terms used in the team’s name, which seems to matter [35]. Fear of the term ‘palliative’ can be explained by common social representation’s associating palliative care with end-of-life care, giving the impression that death is near. It has been reported that families call PPC teams “the angel of death”.

Therefore, the term “paediatric liaison teams”, instead of PPC teams, adopted by the Federal Government through the Royal Decree [22] seems to be more appropriate and efficacious to ensure acceptability of those services among families. Some solutions around this include for the team better communicating to the family what palliative care is and the use of the term Liaison as an alternative.

PLTs provide humane and professional support to families throughout the journey of a child’s illness and death. Quality of relationship is a core element of care, whether for curative or palliative care. Characteristics of this relationship, essential to ensure continuity of care, were found in several theoretical frameworks presented hereafter and linked to the PLT’s activities.

Although Harding et al. highlight the lack of an ideal PPC model, [36] the pop-up model [37] suggests a shared care model of palliative care, where primary health services and specialist PPC providers collaborate. This is the case for the Belgian PLTs, considered mostly as a second line service, which aim is to assist and support the primary health care team (GP and home care nurses). The pop-up model underlines the necessity to provide flexible care.

This coordination between specialists and primary health services can be found in community-based PPC such as reported by Kaye et al. [16]. According to Goldhagen et al. this model was even found to improve health-related quality of life and to reduce hospitalisation utilisation and costs [38].

The elements of continuity of care described by Haggerty, [5] can be achieved through nurse-driven care activities, [39–41] which are demonstrated by the PLTs studied here. Moreover, continuity of care can be rooted in the fundamentals of humanistic nursing theories applied to palliative care, [42–44] which are focused on individualised, holistic and family-centred care.

Those components can by summarised in the paradigm of integrated care suggested by Milstein, [45] which advocates the development of professional attitudes of “being with” families throughout the child’s disease trajectory, both in curative and palliative stages. This constant presence of the team alongside the family can be seen as a form of companionship, and is an important resource of healing. PLTs report that parents express the valuable presence of teams being there for them “day after day”.

This is supported by Weaver et al. [46] and Moonley et al., [47] who strengthen the role of relationships between parents and health care professionals in order to help families to cope with the uncertainty and adversity of their child’s illness, leading to mixed, sometimes dichotomous feelings such as joy and sadness, hope and despair as reported by Rallison [48].

Furthermore, Carter introduces the concept of liminality which is an expression used in anthropology. Liminality refers to the intimate parental experience being nowhere, in a place where usual landmarks are becoming evanescent [49]. This can be the case for parents experiencing isolation when caring for their ill child. Through home visits, active listening and coordination of care, PLTs aim to reach parents where they are, in order for them to feel less isolated and help them reconnect to social life.

Several authors [50, 51] have extrapolated the concept of holding developed by the psychoanalyst D.W. Winnicott [52] as the essence of palliative care, arguing the importance of offering a secure and compassionate environment. At the same time paternalistic or judgmental attitudes should be avoided. Such support is essential for families who may be both vulnerable and isolated. Transposing Winnicott’s concept of the ‘good enough mother’ to the ‘good enough paediatric liaison team’ could offer new perspectives of developing clinical guidelines which best ensure continuity of care.

Following on from the results of and challenges highlighted in our study, several developments of PPC in Belgium are presented hereafter.

### Perspectives

Further discussions and workgroups on how to implement valid indicators of activity, identify children’s palliative stages, and record diseases according to ICD-10 classification are in preparation among the PLT, the Cancer Plan and several authors of this manuscript. This could improve the quality of reporting, align the data collected to international research [53, 54] and strengthen the legitimacy and sustainability of those specialised paediatric liaison teams.

In conjunction with this, constructive national guidelines on the organisation of PPC are currently being prepared by the Belgian Paediatric Palliative Care Group (BPPC) [55]. Started in 2014, the BPPC assembles about 70 actors of PPC in Belgium from different professional backgrounds. The BPPC aims to elaborate guidelines on 5 themes: pain and symptom control, definition of paediatric palliative care, shared decision-making, organization of care, ethics and end-of life care.

It is presumed that better reporting of activities and developing guidelines might partially improve the quality of PPC. Promoting quality in PPC is a potential area of research [56]. This is in line with the EAPC recommendation on outcome measures in palliative care and quality indicators which are currently restricted to studies on adults [57, 58].

Belgian PLTs express strong interest for implementing tools to identify eligible children for PPC, to explore the individual quality of life of children benefitting from PPC, and to measure the impact of care provided. The relevance and feasibility of using several instruments, such as the Paediatric Palliative Screening Scale (PaPas Scale), [59] the direct weighted Scheduled Evaluation of Individual Quality of Life (SEI-QoL-dw) [60] or the Children’s Palliative Outcome Scale (c-POS) was discussed [61] with paediatric liaison teams.

### Limitations

To our knowledge, this is the first study describing the paediatric patients followed in Belgium by the specialised PPC teams, called liaison teams. The activities of these teams, including collaborative practice, are summarised to give a national snapshot of activity of PPC over a 5 year period.

However, the main limitation of the study lies in the fact that access to exclusively aggregated data did not permit further statistical analysis. Furthermore, some definitions in relation to curative and palliative care, reporting of patients with more than one diagnosis, and criteria for when follow-up might be concluded may assist in reducing disparities in reporting between different teams.

## Conclusion

This secondary data analysis shows that Belgian PLTs ensure continuity of care through personalised, integrated care which starts at the time of diagnosis and continues, after a child’s death, through bereavement care. The provision of contact with a PLT in all care settings gives the opportunity to build trusted relationship, which might reduce the important burden of a life-limiting condition on the entire family’s life.

Nevertheless, improvements in data collection, development of national guidelines in PPC and implementation of specific outcome measures in routine clinical care are areas that could be further developed and described in future years.
